# A Retrospective Epidemiological Study of Mortality in a Pediatric Intensive Care Unit: Trends, Causes, and Risk Factors (2014–2023)

**DOI:** 10.3390/children13030337

**Published:** 2026-02-27

**Authors:** Zhiping Zou, Jie Cheng, Xiaoyu Xiong, Dapeng Chen, Liping Tan, Hongdong Li

**Affiliations:** 1 Department of Emergency, Children’s Hospital of Chongqing Medical University, National Clinical Research Center for Children and Adolescents’ Health and Diseases, Ministry of Education Key Laboratory of Child Development and Disorders, Chongqing Key Laboratory of Child Rare Diseases in Infection and Immunity, Chongqing 400014, China; 2 Department of Pediatric Intensive Care Unit, Children’s Hospital of Chongqing Medical University, Chongqing 400014, China; 3 Department of Clinical Laboratory, Children’s Hospital of Chongqing Medical University, Chongqing 400014, China

**Keywords:** mortality, pediatric intensive care unit, epidemiology, risk factor, early death

## Abstract

**Background**: Continuous monitoring of mortality trends and a thorough understanding of death epidemiology in Pediatric Intensive Care Units (PICUs) are critical for healthcare quality assessment, rational resource allocation, and quality improvement initiatives. This study aimed to analyze a decade-long mortality rate, its temporal trends, and the primary causes of death and to elucidate the independent risk factors for early death within a PICU cohort. **Methods:** This retrospective cohort study included all PICU admissions at the Children’s Hospital of Chongqing Medical University from 1 January 2014, to 31 December 2023. Data collection covered demographic characteristics, primary diagnoses, comorbidities, requirements for mechanical ventilation or vasoactive drugs, lengths of stay, and patient outcomes. The multivariate logistic regression analysis was used to determine independent risk factors associated with early death. **Results:** Among 21,910 ICU admissions, the overall mortality rate was 2.3% (n = 512), with a historical range of 0.41% to 5.71%, indicating a gradual decline after 2019. Early mortality (death within 24 h of admission) accounted for 13.9% (71/512) of all deaths. Patients in the early mortality group presented with more severe disease conditions, including shock, sepsis, and postoperative status (*p* < 0.05). Laboratory findings at admission revealed significantly higher procalcitonin and lactate levels, along with lower albumin levels, in this group than in children who died >24 h after admission (*p* < 0.05). Multivariate analysis revealed that a need for invasive mechanical ventilation (OR = 3.03; 95% CI: 1.68–5.58; *p* < 0.001), elevated lactate levels (OR = 1.10; 95% CI: 1.02–1.17; *p* = 0.009), and postoperative status (OR = 0.29; 95% CI: 0.09–0.73; *p* = 0.017) were independent risk factors for early mortality. **Conclusions:** Despite an overall decline in mortality since 2019, early mortality among high-risk patients—such as those requiring invasive mechanical ventilation or those presenting with elevated lactate levels—requires attention. Prompt recognition of these risk profiles and timely intervention are crucial for improving outcomes in children.

## 1. Introduction

The prevention of child mortality constitutes a cornerstone objective for public health interventions worldwide and serves as a critical gauge of a nation’s economic and social development [1]. However, child mortality, particularly deaths occurring within Pediatric Intensive Care Units (PICUs), remains a significant global health challenge [2]. As medical centers that admit the most critically ill children, the mortality rate within PICUs serves as a concentrated reflection of the most complex and severe child health issues within a region [3].

While numerous studies have reported PICU mortality data, these often focus on specific disease entities or are based on information from many years ago. For instance, Punchak et al. reported a mortality rate of 25% in Mozambique in 2013 [4], whereas Moynihan et al. reported a mortality rate of 2.6% across Australia and New Zealand between 2006 and 2016 [5]. In a study by Kirschen et al., 20.7% of patients were declared braindead between 2012 and 2017 [6]. Data from China reported by Wu et al. revealed a mortality rate of 8.9% from 2009 to 2017 [7]. However, a systematic and comprehensive analysis of overall mortality trends and underlying causes in PICUs over the past decade is currently lacking. Significant variations in PICU mortality rates and the distribution of leading causes of death are likely attributable to marked disparities in healthcare resources, technical capabilities, and socioeconomic factors across different regions and countries with varying levels of economic development. Therefore, an in-depth analysis of the epidemiological characteristics, root causes, and changing trends of child deaths in PICUs is crucial for assessing the quality of care in these units, identifying weaknesses in the diagnosis and treatment process, and optimizing resource allocation [5].

Therefore, up-to-date characterization of mortality patterns in PICUs is essential to better understand current disease burden and care outcomes. This study aimed to analyze trends, epidemiological characteristics and causes of mortality among children admitted to the Pediatric Intensive Care Unit of the Children’s Hospital of Chongqing Medical University between 1 January 2014, and 31 December 2023, with a particular focus on early mortality.

## 2. Materials and Methods

### 2.1. Study Design and Patients

This investigation was a retrospective cohort study conducted at the Children’s Hospital of Chongqing Medical University. The study population included all pediatric patients aged between 1 month and 18 years who were admitted to the PICU from 1 January 2014, to 31 December 2023. The exclusion criteria were as follows: (1) neonatal patients (admitted to the NICU) and (2) patients whose clinical data were missing. A standardized data collection form was used to extract information from the electronic medical records (EMR) system. The primary outcome was ICU death. Ethical approval was granted by the hospital’s Institutional Review Board (2023-423), and the need for informed consent was waived given the retrospective study design.

### 2.2. Data Collection

Data regarding deceased children were collected, including demographic characteristics, comorbidities, critical illness status, laboratory parameters, use of vasoactive medications, antimicrobial management, respiratory support, and lengths of hospitalization. The primary mode of death were compared and categorized as death despite maximal support, brain death, and withdrawal of life-sustaining treatment (WLST), among others. Furthermore, the diagnosis of sepsis in this study was based on the Phoenix criteria. During the study period, key operational metrics at the hospital—including staffing, bed capacity, and patient acceptance rates—remained stable.

Laboratory parameters included the following: (1) hematological indices: white blood cell counts, platelet counts, and hemoglobin levels; (2) biochemical markers: albumin, glucose, and lactate levels. Lactate was measured from arterial samples and the first value of lactate at admission was used. (3) inflammatory markers: c-reactive protein (CRP) and procalcitonin (PCT) levels; (4) coagulation profiles: international normalized ratios (INRs) and activated partial thromboplastin time (APTTs); and (5) microbiological data: pathogen culture results.

### 2.3. Statistical Analysis

Continuous variables are presented as medians with interquartile ranges (IQRs), whereas categorical variables are summarized as frequencies and proportions. Group comparisons were performed using the chi-square test or Fisher’s exact test for categorical variables, as appropriate. The Wilcoxon rank-sum test was used for nonnormally distributed continuous variables, and the independent samples *t* test was employed for normally distributed data. Variables with a *p* value < 0.10 in the univariate analysis were included in the multivariate model. Odds ratios (ORs) along with their 95% confidence intervals (CIs) were calculated. All the statistical analyses were conducted using SPSS 26.0 (IBM Corp., Armonk, NY, USA). A two-sided *p* value of less than 0.05 was considered to indicate statistical significance.

## 3. Results

### 3.1. Demographic Characteristics

**Figure 1:**
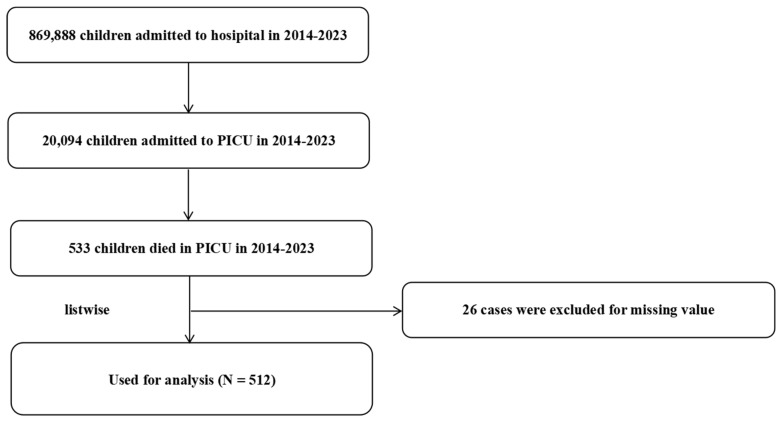
The flowchart of this study.

In this study, an epidemiological analysis of 512 pediatric deaths in the PICU from 2014 to 2023 was conducted. The flow chart of this study is shown in Figure 1. The demographic characteristics of the study participants are presented in Table 1. The results reveal that the median age of deceased children was 1.71 years (IQR = 0.50–6.00), with 57.23% males and 42.77% females (*p* > 0.05). With respect to underlying diseases, congenital heart disease (CHD) was relatively common (16.80%), as were hematologic/oncologic disorders such as myelosuppression after chemotherapy and leukemia, which accounted for 8.79% and 5.66%, respectively. Upon admission, critical conditions were widespread: shock (46.88%), coagulation dysfunction (34.96%), sepsis (32.03%), a history of cardiopulmonary resuscitation (22.07%) and postoperative status (20.90%). These conditions occurred either individually or in combination at the time of admission. Furthermore, 82.03% of the children required vasoactive drugs, and 97.85% received antibiotic therapy, highlighting infection and circulatory failure as significant risk factors for mortality.

### 3.2. Comparisons of Clinical Characteristics Between the Children Who Died After 24 h and Those Who Died Within 24 h

Deaths occurred in 441 children after the first 24 h following admission and in 71 children within 24 h of admission (Table 2). The early mortality group (≤24 h) had a significantly greater incidence of sepsis (52.11% vs. 28.80%, *p* = 0.009) and shock (61.97% vs. 44.44%, *p* < 0.001) than the late mortality group. Additionally, laboratory findings revealed markedly elevated lactate levels (5.00; IQR = 2.40–9.20 vs. 1.80; IQR = 1.00–3.80; *p* < 0.001) and PCT levels (16.29; IQR = 1.14–87.15 vs. 1.63; IQR = 0.21–16.37; *p* < 0.001), along with lower pH (7.27; IQR = 7.16–7.39 vs. 7.38; IQR = 7.29–7.44; *p* < 0.001) and serum albumin levels (26.60; IQR = 21.30–32.80 vs. 31.90; IQR = 27.00–37.70; *p* < 0.001) in the early mortality group versus the late mortality group. In addition, the proportion of patients requiring invasive mechanical ventilation was significantly greater in the early mortality group than in the late mortality group (>24 h) (61.97% vs. 31.29%, *p* < 0.001). Conversely, compared with the early mortality group, the late mortality group had significantly greater incidences of CHD (18.37% vs. 7.04%) and leukemia (6.58% vs. 0.00%) (both *p* < 0.05).

### 3.3. Annual Mortality Trends, Primary Causes of Death, and Variations by Sex

**Figure 2:**
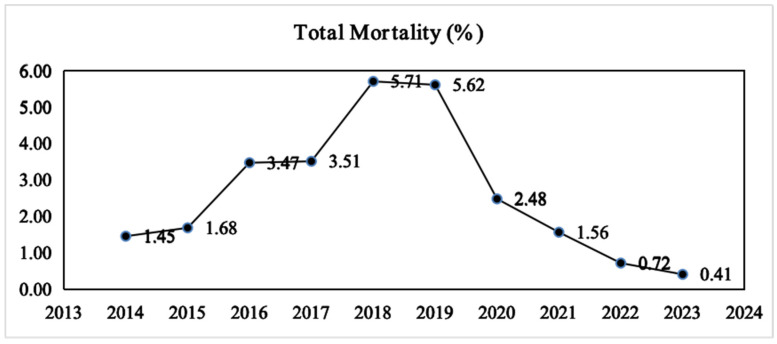
Trend of total mortality rates between 2014 and 2023.

**Figure 3:**
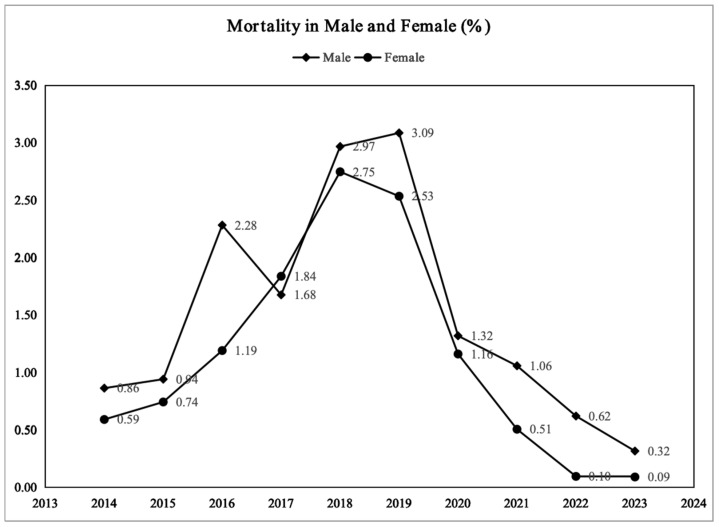
Trend of mortality rates by sex group, 2014–2023.

The overall mortality rate in the PICU demonstrated a consistently increasing trend from 2014 to 2019, increasing from 1.45% to a peak of 5.71% (in 2018), followed by a marked decline starting in 2020, with the rate decreasing to 0.41% by 2023 (Figure 2). This pattern reflects a dynamic, biphasic trend of initial increase followed by a subsequent decrease. Sex-based analysis revealed that the overall mortality rate was higher for male children than for female children, with the disparity being particularly pronounced during the 2016–2019 period, reaching a peak difference of 3.1% versus 2.7%. However, since 2020, mortality rates for both sexes showed a rapid decline, with a more accelerated decrease observed in females. By 2023, the female mortality rate approached zero, while the male mortality rate remained at approximately 0.3% (Figure 3). This suggests that male children may exhibit greater vulnerability in critical care settings.

**Figure 4:**
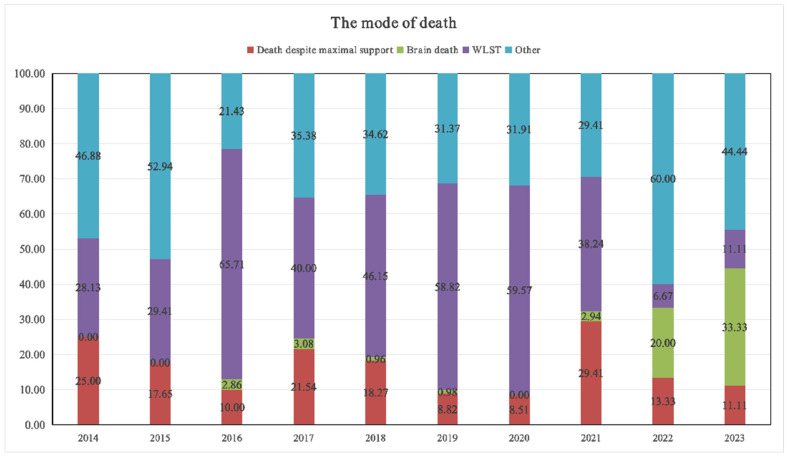
The mode of death in PICU. WLST: withdrawal of life-sustaining treatment. The red, green, purple, and blue sections represent, respectively, death despite maximal support, brain death, WLST, and others.

With respect to the mode of death, WLST remained the predominant factor from 2016 to 2020, accounting for more than 40% of the cases and peaking at 65.71% in 2016 (Figure 4). However, starting in 2021, the proportion of WLST declined significantly. The prevalence of “brain death” increased to 20.00% in 2022 and further increased to 33.33% in 2023, suggesting an increase in the admission of children with neurological diseases or severe brain injuries coupled with poor prognosis. Additionally, the proportion of deaths categorized as “other” rebounded to 44.44% in 2023, possibly involving complex etiologies such as rare diseases or genetic metabolic disorders.

### 3.4. Risk Factors for Early Mortality (≤24 h After Admission)

Table 3 presents the logistic regression analysis of risk factors for early mortality among 512 children in the PICU. Univariate analysis revealed invasive mechanical ventilation, lactate levels, postoperative status, sepsis, shock, coagulation dysfunction, albumin levels, PCT levels, CHD, and pH levels as significant predictors of early mortality (*p* < 0.05). Multivariate analysis confirmed that invasive mechanical ventilation (OR 3.03, 95% CI 1.68–5.58; *p* < 0.001), elevated lactate levels (OR 1.10, 95% CI 1.02–1.17; *p* = 0.009), and postoperative status (OR 0.29, 95% CI 0.09–0.73; *p* = 0.017) were independently associated with early mortality.

## 4. Discussion

This study retrospectively analyzed clinical data related to 512 pediatric deaths in the PICU between 2014 and 2023, aiming to explore their epidemiological characteristics and risk factors for early death (within 24 h of admission) [8,9]. Our findings revealed that the overall mortality rate was 2.3%, with a historical range of 0.41% to 5.71%, indicating a gradual decline after 2019. Invasive mechanical ventilation, elevated lactate levels and postoperative status were independent risk factors for early mortality, highlighting important implications for identifying high-risk children and optimizing clinical intervention strategies.

In this study, the overall mortality rate in the PICU was 2.3%, which was similar to that reported in previous studies [5,10,11,12] but lower than the 8.9% reported by Wu [7]; another single-center survey performed in Guangzhou between 1 January 2009, and 31 December 2017, suggested that regional differences in healthcare resources, treatment protocols, and patient populations may have contributed to the observed disparities. This underscores the need for standardized guidelines and best practices to ensure equitable and high-quality care for critically ill children across different settings. Our results also demonstrated that the mortality rate decreased significantly over these 10 years, which was consistent with the findings of other studies [11,13]. The observed reduction in mortality is likely attributable to multiple factors. First, improvements in the quality of PICU treatment over time have been documented, reflecting a trend toward steady advancement [14]. Second, changes in admission criteria, which now include less severe cases, and a shift toward end-of-life care at home have contributed to the reduction in mortality [15,16,17]. A nationwide study conducted between 1989 and 2003 revealed a significant upward annual trend in home death among patients with complex chronic conditions [18]. Finally, global fertility rates have been declining for decades, a trend mirrored in China [19]. Despite the gradual relaxation of population policies over the past ten years, this decline has persisted, culminating in China’s entry into negative population growth in 2022 [20].

Underlying illness often contributes to pediatric mortality [20]. Our results revealed that CHD (16.80%) was the most common comorbidity identified in this study, followed by hematologic/oncologic conditions such as myelosuppression after chemotherapy (8.79%) and leukemia (5.66%). This distribution aligns with the findings of previous research indicating that structural heart disease and chronic underlying conditions are high-risk factors for mortality in critically ill children [21,22]. Moreover, the use of invasive mechanical ventilation (OR 3.03, 95% CI 1.68–5.58; *p* < 0.001) emerged as a significant risk factor for early mortality in our study. These findings are in line with previous research indicating that children requiring mechanical ventilation, especially those with prolonged ventilation needs, face an increased risk of adverse outcomes [23]. The association between elevated lactate levels (OR 1.10, 95% CI 1.02–1.17; *p* = 0.009) and early mortality also aligns with the findings of previous reports, suggesting that metabolic derangements play a crucial role in the prognosis of critically ill children [24]. Lactate as a marker of disease severity at admission versus a potentially modifiable target through early resuscitation. In addition, compared to the lactate values at admission, 12 h after admission with lactate clearance 12 h after admission enhance the prediction capacity of outcomes [25]. In this study, we only included the initial lactate results and overlooked the dynamic changes in lactate, which will be the focus of our future efforts.

Interestingly, postoperative status (OR 0.29, 95% CI 0.09–0.73; *p* = 0.017) was a protective factor against early mortality in our analysis. This could be attributed to the complex physiological state of children following surgery, who may require more intensive monitoring and care [26]. Our results underscore the importance of close postoperative surveillance and prompt intervention to mitigate the risk of early mortality in this vulnerable population. However, this finding of delayed mortality was observed in deceased patients. Postoperative status may also lead to later deaths, including planned complex surgeries that are initially stable but develop complications later, or deaths after prolonged support rather than early collapse.

In our study, we also found that the critical illness status of these patients in the PICU was shock (46.88%), followed by coagulation dysfunction (34.96%), sepsis (32.03%), a history of cardiopulmonary resuscitation (22.07%) and postoperative status (20.90%). The high incidence of shock as a cause of death may be related to the severity of illness in PICU patients, as shock often represents a critical deterioration in a child’s condition, leading to multiple organ dysfunction and eventually death. Coagulation dysfunction, closely associated with shock, can further exacerbate a patient’s condition by causing bleeding or thrombosis, thereby increasing mortality risk. Sepsis, another significant cause of death, is often a complication of severe infections in critically ill children, highlighting the importance of timely and effective infection control measures [27]. A history of cardiopulmonary resuscitation indicates that a patient has already experienced a life-threatening event, and their subsequent survival is often compromised by underlying illness and the physical stress of resuscitation [28]. While postoperative status serves as a protective factor against early death, it remains a risk factor for overall mortality, potentially reflecting surgical complications and highlighting the need for meticulous postoperative care and monitoring in the PICU [26].

WLST was the predominant mode of death through the 2016–2020 period, accounting for more than 40% of the cases. Notably, in 2016, the proportion peaked at 65.71% but significantly decreased in 2022 and 2023, reflecting the ethical decisions some families made when confronted with irreversible medical conditions. This shift may indicate an evolving understanding and acceptance of end-of-life care among families and healthcare providers. The decrease in treatment withdrawal cases in recent years could also be attributed to improved communication between medical teams and families, leading to more informed decisions that prioritize the child’s quality of life. Additionally, advancements in palliative care services may have provided families with alternative options to manage their child’s terminal illness, reducing the perceived need for treatment withdrawal [29,30].

The proportion of children diagnosed with brain death in our study increased significantly in 2022 and 2023. This increase may be attributed to widespread influenza outbreaks during those years, which can lead to influenza-related encephalopathy [31,32]. The heightened incidence of brain death diagnoses could also reflect improved diagnostic criteria and increased awareness among medical professionals, leading to more accurate and timelier identification of brain death in pediatric patients. Furthermore, influenza outbreaks may exacerbate underlying health conditions in vulnerable children, increasing their susceptibility to severe neurological complications and subsequent brain death.

The strengths of this study lie in its comprehensive analysis of early mortality risk factors and causes of death in PICU patients, utilizing a robust dataset that enhances the reliability of our findings. However, this study has several limitations. First, the study was conducted at a single center, which may limit the generalizability of our results to other healthcare settings with different patient populations and clinical practices. Second, the retrospective nature of the study design introduces potential biases, such as selection bias and information bias, which could affect the accuracy of our findings. Third, although we attempted to control for confounding variables, there may still be unmeasured factors that could influence the relationship between the identified risk factors and early mortality. Last, whether delayed referral and pre-PICU management impact early mortality requires elucidation. Future research should aim to address these limitations by conducting multicenter, prospective studies with larger sample sizes to validate our findings and explore additional risk factors and causes of death among PICU patients. Additionally, further studies are needed to investigate the underlying mechanisms of the identified risk factors and develop targeted interventions to improve the prognosis of critically ill children.

## 5. Conclusions

In conclusion, mortality in critically ill children improved during the study period, with a notable decrease in the proportion of cases involving WLST. This improvement may be attributed to enhanced communication between medical teams and families, leading to more informed and patient-centered decisions. Additionally, invasive mechanical ventilation and elevated lactate levels were identified as significant risk factors for early mortality in critically ill children, emphasizing the need for close monitoring and prompt intervention in PICU settings. The increase in the number of brain death diagnoses during influenza outbreaks underscores the importance of preventive measures and early recognition of severe neurological complications.

## Figures and Tables

**Table 1:** Characteristics of 512 children who died in Intensive Care Unit (ICU).

Characteristics	Total (n = 512)
Demographic data	
Age (years) (median, IQR)	1.71 (0.50–6.00)
Sex (male) (n, %)	293 (57.23%)
Place of Origin	
Rural (n, %)	294 (57.42%)
Urban (n, %)	218 (42.58%)
Comorbidities	
Hematologic/Oncologic disorders	
Myelosuppression after chemotherapy (n, %)	45 (8.79%)
Leukemia (n, %)	29 (5.66%)
Tumor (n, %)	20 (3.90%)
Lymphoma (n, %)	8 (1.56%)
Aplastic anemia (AA) (n, %)	6 (1.17%)
Hemophagocytic lymphohistiocytosis (HLH) (n, %)	6 (1.17%)
Congenital/Genetic disorders	
Congenital heart diseases (CHD) (n, %)	86 (16.80%)
Metabolic diseases (n, %)	17 (3.32%)
Chromosomal abnormality (n, %)	10 (1.95%)
Global developmental delay (n, %)	4 (0.78%)
Anatomical abnormalities	
Airway developmental abnormalities (n, %)	12 (2.34%)
Digestive tract malformations (n, %)	10 (1.95%)
Bronchopulmonary dysplasia (BPD) (n, %)	7 (1.37%)
Immune/rheumatologic disorders	
Primary immunodeficiency (PID) (n, %)	22 (4.30%)
Rheumatic immune diseases (RID) (n, %)	9 (1.76%)
Critical illness status	
Shock (n, %)	240 (46.88%)
Coagulation dysfunction (n, %)	179 (34.96%)
Sepsis (n, %)	164 (32.03%)
History of cardiopulmonary resuscitation (CPR) (n, %)	113 (22.07%)
Postoperative status (n, %)	107 (20.90%)
Laboratory tests	
Blood routine examinations	
White blood cells (WBC) (10^9/L) (median, IQR)	10.13 (4.34–17.55)
Platelet (PLT) (10^9/L) (median, IQR)	194 (66–354)
Hemoglobin (Hb) (g/L) (median, IQR)	99 (8–116)
Inflammatory indicators	
C-reactive protein (CRP) (mg/L) (median, IQR)	8.00 (8.00–35.00)
Procalcitonin (PCT) (ng/mL) (median, IQR)	2.08 (0.23–21.48)
Blood gas analysis	
Potential of hydrogen (pH) (median, IQR)	7.37 (7.28–7.43)
Lactate (Lac) (mmol/L) (median, IQR)	2.00 (1.10–4.52)
Albumin (Alb) (g/L) (median, IQR)	31.35 (26.00–37.10)
Length of stay (days) (median, IQR)	8.00 (3.00–17.25)
Requiring for vasoactive drugs (n, %)	420 (82.03%)
Blood purification therapy (n, %)	141 (27.54%)
Antimicrobial managements	
Administrated with antibiotics (n, %)	501 (97.85%)
Administrated with 2 or more classes antibiotics (n, %)	121 (23.63%)
Respiratory support	
Invasive mechanical ventilation (n, %)	182 (35.55%)
Continuous positive airway pressure (CPAP) (n, %)	21 (4.10%)
Death within 24 h (n, %)	71 (13.87%)

**Table 2:** Comparisons of clinical characteristics between the children died after 24 h and died within 24 h.

Characteristics	Died > 24 h (n = 441)	Died Within 24 h (n = 71)	*p*-Value
Demographic data			
Age (years) (median, IQR)	1.67 (0.50–6.00)	2.00 (0.63–5.00)	0.500
Sex (male) (n, %)	254 (57.60%)	39 (54.93%)	0.770
Place of Origin			0.850
Rural (n, %)	252 (57.14%)	42 (59.15%)	
Urban (n, %)	189 (42.86%)	29 (40.85%)	
Comorbidities			
Hematologic/Oncologic disorders			
Myelosuppression after chemotherapy (n, %)	43 (9.75%)	2 (2.82%)	0.091
Leukemia (n, %)	29 (6.58%)	0 (0.00%)	0.023 *
Tumor (n, %)	19 (4.31%)	1 (1.41%)	0.337
Lymphoma (n, %)	8 (1.81%)	0 (0.00%)	0.607
AA (n, %)	5 (1.13%)	1 (1.41%)	0.594
HLH (n, %)	6 (1.36%)	0 (0.00%)	1.000
Congenital/Genetic disorders			
CHD (n, %)	81 (18.37%)	5 (7.04%)	0.028 *
Metabolic diseases (n, %)	14 (3.17%)	3 (4.23%)	0.718
Chromosomal abnormality (n, %)	7 (1.59%)	3 (4.23%)	0.149
Global developmental delay (n, %)	2 (0.45%)	2 (2.82%)	0.094
Anatomical abnormalities			
Airway developmental abnormalities (n, %)	12 (2.72%)	0 (0.00%)	0.388
Digestive tract malformations (n, %)	9 (2.04%)	1 (1.41%)	1.000
BPD (n, %)	7 (1.59%)	0 (0.00%)	0.601
Autoimmune disorders			
PID (n, %)	21 (4.76%)	1 (1.41%)	0.340
RID (n, %)	8 (1.81%)	1 (1.41%)	1.000
Critical illness states			
Shock (n, %)	196 (44.44%)	44 (61.97%)	0.009 *
Coagulation dysfunction (n, %)	147 (33.33%)	32 (45.07%)	0.073
Sepsis (n, %)	127 (28.80%)	37 (52.11%)	<0.001 *
History of CPR (n, %)	100 (22.67%)	13 (18.31%)	0.503
Postoperative status (n, %)	102 (23.13%)	5 (7.04%)	0.003 *
Laboratory tests			
Blood routine examinations			
WBC (10^9^/L) (median, IQR)	10.03 (4.64–17.59)	10.52 (3.61–15.94)	0.635
PLT (10^9^/L) (median, IQR)	196 (68–362)	140 (47–305)	0.111
Hb (g/L) (median, IQR)	99 (81–116)	96 (80–121)	0.841
Inflammatory indicators			
CRP (mg/L) (median, IQR)	8.00 (8.00–33.00)	10.00 (8.00–52.50)	0.171
PCT (ng/mL) (median, IQR)	1.63 (0.21–16.37)	16.29 (1.14–87.15)	<0.001 *
Blood gas analysis			
pH (median, IQR)	7.38 (7.29–7.44)	7.27 (7.16–7.39)	<0.001 *
Lac (mmol/L) (median, IQR)	1.80 (1.00–3.80)	5.00 (2.40–9.20)	<0.001 *
Alb (g/L) (median, IQR)	31.90 (27.00–37.70)	26.60 (21.30–32.80)	<0.001 *
Requiring for vasoactive drugs (n, %)	367 (83.22%)	53 (74.65%)	0.114
Blood purification therapy (n, %)	122 (27.66%)	19 (26.76%)	0.988
Antimicrobial managements			
Administrated with antibiotics (n, %)	432 (97.96%)	69 (97.18%)	0.655
Administrated with 2 or more classes antibiotics (n, %)	100 (22.68%)	21 (29.58%)	0.263
Respiratory support			
Invasive mechanical ventilation (n, %)	138 (31.29%)	44 (61.97%)	<0.001 *
CPAP (n, %)	19 (4.31%)	2 (2.82%)	0.753

AA = aplastic anemia, HLH = hemophagocytic lymphohistiocytosis, CHD = congenital heart diseases, BPD = bronchopulmonary dysplasia, PID = primary immunodeficiency, RID = rheumatic immune diseases, CPR = cardiopulmonary resuscitation, WBC = white blood cells, PLT = platelet, Hb = hemoglobin, CRP = c-reactive protein, PCT = procalcitonin, pH = potential of hydrogen, Lac = lactate, Alb = Albumin, CPAP = continuous positive airway pressure. * with statistical significance, *p* < 0.05.

**Table 3:** Logistic regression analysis of risk factors of early mortality among 512 children in PICU.

Variables	Univariate Analysis			Multivariate Analysis		
	OR	95% CI	*p*	OR	95% CI	*p*
Invasive mechanical ventilation (n, %)	3.58	2.14–6.08	<0.001 *	3.03	1.68–5.58	<0.001 *
Lac (mmol/L) (median, IQR)	1.16	1.10–1.22	<0.001 *	1.10	1.02–1.17	0.009 *
Postoperative status (n, %)	0.25	0.09–0.58	0.004 *	0.29	0.09–0.73	0.017 *
Sepsis (n, %)	2.69	1.62–4.49	<0.001 *			
Shock (n, %)	2.04	1.23–3.44	0.007 *			
Coagulation.dysfunction (n, %)	1.64	0.98–2.72	0.003 *			
Alb (g/L) (median, IQR)	1.06	1.02–1.10	<0.001 *			
PCT (ng/mL) (median, IQR)	1.02	1.01–1.02	<0.001 *			
CHD (n, %)	0.34	0.12–0.79	0.023 *			
Myelosuppression after chemotherapy (n, %)	0.27	0.04–0.90	0.073			
pH (median, IQR)	0.01	0.00–0.06	<0.001 *			

Lac = lactate, Alb = Albumin, PCT = procalcitonin, CHD = congenital heart diseases, pH = potential of hydrogen, * with statistical significance, *p* < 0.05.

## Data Availability

The data that support the findings of this study are available upon reasonable request from the corresponding author. The data are not publicly available due to ethical reasons.

## Abbreviations

The following abbreviations are used in this manuscript:

PICUs	Pediatric Intensive Care Units
EMRs	Electronic medical records
MODS	Multiple organ dysfunction syndrome
WLST	Withdrawal of life-sustaining treatment
IQR	Interquartile ranges
OR	Odds ratio
CIs	Confidence intervals
CHD	Congenital heart disease
CRP	C-reactive protein
PCT	Procalcitonin
AA	Aplastic anemia
HLH	Hemophagocytic lymphohistiocytosis
BPD	Bronchopulmonary dysplasia
PID	Primary immunodeficiency
RIDs	Rheumatic immune diseases
CPR	History of cardiopulmonary resuscitation
WBCs	White blood cells
PLT	Platelet
Hb	Hemoglobin
pH	Potential of hydrogen
Lac	Lactate
Alb	Albumin
CPAP	Continuous positive airway pressure
